# A register-based approach to identifying treatment-resistant depression—Comparison with clinical definitions

**DOI:** 10.1371/journal.pone.0236434

**Published:** 2020-07-30

**Authors:** David Hägg, Philip Brenner, Johan Reutfors, Gang Li, Allitia DiBernardo, Robert Bodén, Lena Brandt

**Affiliations:** 1 Karolinska Institutet, Centre for Pharmacoepidemiology, Karolinska University Hospital, Stockholm, Sweden; 2 Janssen, Global Services, Titusville, New Jersey, United States of America; 3 Department of Neuroscience Psychiatry, Uppsala University, Uppsala, Sweden; National Institutes of Health, UNITED STATES

## Abstract

**Background:**

Several definitions of treatment-resistant depression (TRD) are used for clinical research, but no verified model for use in register data exists. We aimed to compare a novel model created for use in register data—the Karolinska Institutet Model (KIM)–to the clinical definitions regarding the proportion of patients identified with TRD, their characteristics and clinical outcomes.

**Methods:**

All patients in Sweden initiating antidepressant treatment with a diagnosis of depression in specialized healthcare 2006–2014 were identified and followed in national registers. In KIM, patients who initiated a third sequential, >28-day antidepressant treatment trial were defined as having TRD. Proportion of TRD and patient characteristics were compared with register adaptations of the European Staging Model (ESM), Massachusetts General Hospital Staging Method (MGH-s), and Maudsley Staging Model (MSM). Differences in patient characteristics were assessed with Chi-square tests and one-way ANOVAs. Hazard ratios for psychiatric hospitalization and for death from external causes were estimated by Cox proportional hazard regressions.

**Results:**

Out of 127,108 antidepressant initiators with depression, the highest proportion of TRD was found using the MGH-s (19.0%), followed by MSM (15.3%), KIM (12.9%), and ESM (9.5%). Clinical characteristics were similar across the models. Compared with TRD patients identified by KIM, those identified by ESM had a marginally higher risk for psychiatric hospitalization (adjusted hazard ratio [aHR] 1.03, 95%CI 1.00–1.05), whereas those identified by MGH-s (aHR 0.92; 0.90–0.94) and MSM (aHR 0.95; 0.94–0.97) had a slightly reduced risk. Patients identified by MGH-s showed a reduced mortality compared with KIM (aHR 0.84; 0.72–0.98).

**Conclusions:**

This study provides insight into the differing characteristics of patients captured by various TRD models when used for register research. Models yielding lower proportions of TRD seemed to identify patients with greater morbidity. The KIM may be useful for register based research in TRD.

## 1. Background

In clinical trials on depression intended to emulate clinical practice, the cumulative proportion of patients who do not achieve adequate treatment response after two antidepressant treatment trials is estimated to be up to 50%—commonly referred to as treatment-resistant depression (TRD) [1, 2]. Several definitions and staging methods for TRD have emerged during the last decades, among them the European Staging Method (ESM), the Massachusetts General Hospital Staging Model (MGH-s), and the Maudsley Staging Model (MSM) [3–6]. These models share the general criterion of two sequential failed antidepressant treatment trials of adequate length and duration, but vary regarding other parameters such as type of drug treatment required, treatment episode length required, or requirement of increasing the antidepressant dose (Panel 1).

**Panel 1:** Overview of the characteristics of the Karolinska Institutet model (KIM), the European Staging Method (ESM), the Massachusetts General Hospital Staging Method (MGH-s), and the Maudsley Staging Method (MSM).

		KIM	ESM	MGH-s	MSM
**Original version**	***TRD threshold or TRD severity points***	TRD threshold	TRD threshold	TRD severity points (Total score = summation of each treatment trial)	TRD severity points (Total score = 3 to 15)
	***Number of treatment failures to be defined as TRD***	2	2	1 point per nonresponsive AD treatment episode	Level 1: 1–2 ADs = 1 point(s);
					Level 2: 3–4 ADs = 2;
					Level 3: 5–6 ADs = 3;
					Level 4: 7–10 ADs = 4;
					Level 5: >10 ADs = 5;
	***Adequate length of treatment episode/dose***	At least 28 days	NA	See S1 Table for definition of adequate dose	NA
	***Required duration***	NA	TRD1–12- to 16-weeks	At least 6 weeks	Acute (≤365 days) = 1 point^a^
			TRD2–18- to 24-weeks duration		Subacute (13 to 24 months) = 2 points^a^
					Chronic (>24 months) = 3 points^a^
			TRD3–24- to 32-weeks duration		
			TRD4–30- to 40-weeks duration		
			TRD5–36- to 52-weeks duration		
	***Augmentation***	NA	NA	0.5 point for augmentation	1 point
	***Optimisation of dose***	NA	NA	0.5 point for optimisation	NA ^c^
	***ECT/rTMS***	Counted as a treatment episode	NA	3 point for ECT	1 point for ECT
	***Symptom severity***	NA	NA	NA	Subsyndromal^b^ = 1 point
					Mild = 2 points
					Moderate = 3 points
					Severe without psychosis = 4points
					Severe with psychosis = 5 points
**Adapted version for register data**	***TRD threshold or TRD severity points***	Unchanged	Unchanged	TRD threshold (TRD definition threshold = total score ≥4 points)	TRD threshold (TRD definition threshold = total score ≥5)
	***Number of treatment failures to be defined as TRD***	Unchanged	Unchanged	Nonresponse to each adequate (at least six weeks at an adequate dose) trial of an AD (1 point per trial)–See S1 Table for definition of adequate dose.	Treatment failures:
					1–2 ADs = 1 point
					3–4 ADs = 2 points
					5–6 ADs = 3 points
					7–10 ADs = 4 points
					>10 ADs = 5 points
	***Adequate length of treatment episode/dose***	Unchanged	At least 28 days	See S1 Table for definition of adequate dose.	At least 28 days
	***Required duration***	Unchanged	12- to 16-week	Unchanged	NA
	***Augmentation***	Unchanged	Unchanged	Unchanged	Unchanged
	***Optimisation of dose***	Unchanged	Unchanged	Unchanged	NA ^c^
	***ECT/rTMS***	Unchanged	Unchanged	Unchanged	Unchanged
	***Symptom severity***	Unchanged	Unchanged	Unchanged	ICD-10: Major depressive disorder:
					F32.0 and F33.0 mild = 2 points
					F32.1 and F33.1 moderate = 3 points
					F32.2 and F33.2 severe without psychotic features = 4 points
					F32.3 and F33.3 severe with psychotic features = 5 points

^a^ Duration cannot be used as in the original version since we have a one-year follow-up.

^b^ Information not available in registers.

^c^ Information on optimisation of dose, optimisation of duration not available in registers.

AD = Antidepressant; ECT = Electroconvulsive therapy; repetitive transcranial magnetic stimulation; rTMS = repetitive transcranial magnetic stimulation; TRD = treatment-resistant depression; CRD = chronic resistant depression; ICD = International Statistical Classification of Diagnoses; NA = not applicable.

Although fundamentally a clinical concept based on the lack of remission of symptoms, research questions regarding rare or long-term characteristics of patients with TRD warrant large study populations and/or long follow-up time, which may be difficult to achieve in clinical studies. For this purpose, several studies identifying patients with TRD in administrative databases and health care registers have been performed: in US claims data for health economic analyses [7–10], in the UK General Practice Research Database [11], the National Health Insurance Database in Taiwan [12], and in Swedish health and population registers [13, 14]. One study applied a register adaptation of the MGH in data from the US Veterans Health Administration [15].

Several of the parameters used in the clinical definitions may be difficult to adapt to register research due to absence of clinical information, e.g. on type and duration of symptoms. Instead, a common method has been to solely define the TRD group as patients with a registered diagnosis of depression and initiation of at least a third sequential treatment trial within a specified time-frame—an approach supported by recent suggestions on how to operationalize TRD in an evidence-based manner [16]. However, register based methods as well have varied substantially regarding parameters such as inclusion/exclusion criteria and treatment episode type and length. The data-bases also vary in type and granularity of both patient and prescription/claims data available for study. The proportion of patients identified with TRD among patients with a major depressive disorder (MDD) in the register based studies so far ranges from 6.6% [10] to 29% [8], which is far below the up to 50% seen in clinical studies [1, 2].

The aims of this study were to compare an algorithm for identifying patients with TRD for use in health registers—the Karolinska Institutet Model (KIM)–regarding the proportion and characteristics of patients identified with register adaptations of the ESM, the MGH-s, and the MSM, and to compare the patients identified in the different models regarding risk for the clinical outcomes of psychiatric hospitalization and death from external causes, i.e. accidents and suicide.

## 2. Method

### 2.1. Data sources

Swedish national healthcare and population registers were used to identify the study cohort, covariates, and outcomes. The Prescribed Drug Register (PDR) contains data on all prescription fills of prescribed drugs in Sweden since July 1^st^, 2005 [17]. The National Patient Register (NPR) contains data on all diagnoses and procedures according to the International Statistical Classification of Diagnoses (ICD) with complete national coverage for Swedish in-patient care since 1987, and for out-patient specialized care since 2001 [18]. The Total Population Register (TPR) contains data on residents in Sweden including place of residence and migration [19]. The Longitudinal Integration Database for Health Insurance and Labour Market Studies (LISA) is a population-based register containing various sociodemographic variables including income and country of birth [20]. The Swedish Cause of Death Register (CDR) contains dates and causes of death, as stated on the death certificate by the signing physician, for all Swedish residents since 1961 [21]. Linkage between data sources was possible using the subjects’ unique personal identity numbers.

### 2.2. Study participants

All patients in Sweden aged 18 years or older with a filled prescription of an antidepressant (ATC-code N06A) between July 1, 2006 and December 31, 2014 (index prescription), and who had a diagnosis of depression (ICD-10 F32: Major depressive disorder, single episode, F33: Major depressive disorder, recurrent or F34: Persistent mood disorders) in the NPR within a time interval of 30 days before, and up to 365 days after, the filled prescription were included in the depression cohort.

To identify treatment initiators, only the patients with no record of using the following treatments during the 180 days preceding the start of the antidepressant episode were eligible for inclusion: antidepressants, lithium, antipsychotics, valproate, lamotrigine, carbamazepine, electroconvulsive treatment (ECT) and repetitive transcranial magnetic stimulation (rTMS). To reduce the risk of diagnostic misclassification, patients with a history of psychosis, mania, bipolar disorder or dementia were excluded, as were patients who were not residents in Sweden for 180 days preceding the first antidepressant prescription fill during the study period.

Patients were followed regarding changes in antidepressant treatment regimens for 365 days after the first prescription fill. The prescribed dose was extracted by semi-manually condensing the prescription text data to shorter form and manually converting it into number of tablets prescribed per day [22]. Taking the prescribed dose and the amount of dispensed drugs into account, the duration of each filled prescription was estimated. The patient was assumed to have discontinued the treatment after 28 days without supply of the drug; i.e., a medication gap.

### 2.3. Definitions of TRD and register adapted versions

#### 2.3.1. The Karolinska Institutet Model (KIM)

According to the KIM, patients were classified with TRD if at least two subsequent treatment episodes were recorded after the first antidepressant treatment. Subsequent treatment episodes could consist of a prescription fill of another antidepressant than the first one (i.e. another ATC-code), a prescription fill of an add-on medication, or administered treatment with ECT or rTMS. Potential add-on medications were identified as lithium, aripiprazole, olanzapine, risperidone, and quetiapine [23, 24]. A treatment episode had to last at least 28 days to be considered an adequate treatment trial. If a prescription for another substance was filled or ECT/rTMS-treatment was initiated sooner, the first treatment was not considered an adequate treatment trial. If a new treatment was started within 14 days after the start of a prior treatment episode which continued it was regarded as part of a combination therapy. Patients were reclassified from non-TRD to TRD from the first day of the third treatment trial.

For patients who filled a prescription of an antidepressant or an add-on medication during ongoing hospitalization, the treatment episode was considered to start on the day of hospital discharge. For patients who were hospitalized after the first antidepressant prescription fill, the assumed duration of the prescription was prolonged with the number of days equivalent to the length of hospitalization. If hospitalization occurred during a gap in medication, the gap was shortened by the number of days in hospital.

#### 2.3.2. The European Staging Method (ESM)

*Original version*. According to the ESM, the patient is classified as either a non-responder (patients who fail to respond to one treatment regimen), with TRD, or with chronic resistant depression (CRD). The categories of non-responders and CRD were not investigated further in this study. The ESM requires that at least two failed consecutive treatments steps of two different drug classes should be classified as the milder level of resistance, and that the duration of each consecutive treatment step should be at least 6–8 weeks. The ESM does not consider add-on treatments, combination therapies, or ECT [3].

*Adapted version*. Resistance to two or more antidepressants of different classes for at least 12- to 16 weeks (ESM: TRD1) was considered to be sufficient to be classified as a TRD patient according to ESM (see Panel 1). ESM was then applied by defining TRD as having two six-week periods of being treated with drugs from two different classes, with a subsequent third initiation of treatment.

#### 2.3.3. Massachusetts General Hospital Staging Model (MGH-s)

*Original version*. MGH-s states that TRD should be defined along a continuous variable of assessing the resistance severity, where each treatment receives 1 score, any add-on treatments or optimizations 0.5 scores, and ECT 3 scores. Time spent in treatment should be at least 6 weeks, and doses should be adequate according to standard specifications [4].

*Adapted version*. Patients received one point for each antidepressant medication for which they obtained at least a 6-week supply at an “adequate dose for treatment of depression” (S1 Table). The adequate dose was assessed for each of the study drugs by a psychiatrist in the research group combining prescription guidelines with scientific evidence. An additional 0.5 points was given if any add-on agent (see above) was added to an antidepressant. Patients who received ECT were given an additional 3 points. An MGH score exceeding 3 points was considered a TRD definition threshold. This threshold corresponds to two adequate antidepressant trials with one optimization strategy each (although other combinations are possible) [25, 26]. Any additional optimization strategy, or an adequate trial of a third antidepressant, would increase the MGH score and thus meet the definition threshold for TRD (see Panel 1).

#### 2.3.4. The Maudsley Staging Model (MSM)

*Original version*. The duration is categorized as acute (≤12 months), sub-acute (13–24 months) or chronic (> 24 months), and yields 1, 2 and 3 points respectively.

MSM takes into account the duration of the current treatment episode and symptom severity. Treatment failures are classified into five levels, from level 1 with 1–2 antidepressants to level 5 with >10 medications. MSM conceptualizes TRD as a continuous variable, where scores of 3–6 represent mild treatment resistance, 7–10 moderate, and 11–15 severe, with points also given for ECT and add-on treatment.

*Adapted version*. In the adaptation of MSM, the criterion for duration of treatment could not be applied, because of our time limit of one-year follow-up. An adequate treatment episode was therefore defined to be at least 28 days. The symptom severity was identified through the fourth position in the ICD-10 diagnoses of MDD, where F32.0 and F33.0 was classified as mild (2 points), F32.1 and F33.1 as moderate (3 points), F32.2 and F33.2 as severe without psychotic features (4 points) and F32.3 and F33.3 as severe with psychotic features (5 points). Patients who received ECT received one point and one additional point if a drug was added to an antidepressant. The number of failed antidepressant treatments were categorized according to the original scheme as level 1: 1–2 ADs = 1 point, level 2: 3–4 ADs = 2 points, level 3: 5–6 ADs = 3 points, level 4: 7–10 ADs = 4 points, and level 5: >10 ADs = 5 points. Since MSM is not designed to classify patients as having TRD but to measure the severity of treatment-resistant depression, we chose to set the definition threshold of TRD to a total score of ≥5, in order to capture the milder cases of patients with severe resistance of antidepressant treatment (see Panel 1).

### 2.4. Covariates and outcomes

Age at the time of entry into the depression cohort was categorized into four categories: 18–29, 30–59, 60–79 and ≥80 years. Annual income in relation to the annual income distribution in Sweden was categorized into: low (bottom 20% of the income distribution), middle, and high (top 20%). Data on country of birth was categorized as follows: Sweden, other Nordic countries, other European countries and other. A Charlson comorbidity index score (CCI) [27], measuring severity of somatic comorbidity, was constructed for all patients using data from the NPR. The number of days until fulfilling criteria for TRD was calculated from the time of entering the MDD cohort.

Data on any psychiatric hospitalization, defined by the ICD-10 codes F00-F99 during the 365 days after being identified with TRD was added to all patients identified with TRD in the four models. Data on mortality from external case of death, defined by the ICD-10 codes V01-Y98 stated as cause of death, was identified in the CDR.

### 2.5 Statistical analysis

P-values were calculated for proportion of TRD and all covariates comparing the four models. Time to psychiatric hospitalization and mortality from an external case of death was analysed by multiple Cox regression adjusted by sex and age. Patients were censored at death or emigration. Associations between the four TRD definitions and outcomes were assessed by hazard ratios (HR) with 95% confidence intervals (CI). Further analyses were done to evaluate if any interactions existed between the four TRD models and the selected covariates on psychiatric hospitalization and mortality from an external case of death. Statistical analyses were performed in SAS version 9.3.

### 2.6 Ethics approval and consent to participate

The study was approved by the Research Ethics Committee at Karolinska Institutet, Stockholm (no. 2017/1236-31/2). Consent was not obtained as the study was based on registry information.

## 3. Results

Descriptive data of the patients categorized according to the four models are shown in Table 1. The study cohort consisted of 127,108 patients (58.4% females) with a diagnosis of depression and who had initiated treatment with an antidepressant. During the one-year follow-up, KIM classified 12.9% of the patients as having TRD. Using the adapted clinical models for comparison the highest proportion of patients classified as having TRD was found by the MGH-s with 19.0%, followed by the MSM with 15.3%, and the ESM with 9.5%.

**Table 1:** Patient characteristics of the total cohort of major depressive disorder (MDD) and the patient groups established by the TRD definitions: Karolinska Institutet Model (KIM), European Staging Method (ESM), Massachusetts General Hospital Staging Method (MGH-s), and Maudsley Staging Method (MSM).

		Anti-depressant initiator and MDD	KIM	ESM	MGH-s	MSM	p-value
	n patients / n TRD patients	127,108	16,453	12,059	24,112	19,486	
	% TRD patients		12.9	9.5	19.0	15.3	<0.001^c^
Females (%)		58.4	58.1	58.0	58.1	58.0	0.995^c^
Age in years	18–29	34.2	29.7	29.0	31.5	30.8	<0.001^d^
	30–59	51.9	53.7	53.3	53.1	53.1	
	60–79	11.6	13.9	14.8	12.8	13.5	
	≥80	2.4	2.7	2.9	2.6	2.6	
Age in years, mean (SD)			41.9 (17.1)	42.4 (17.3)	41.1 (17.1)	41.5 (17.1)	
Income (%)^a^	Low	52.2	51.1	49.9	51.8	51.3	0.049^c^
	Average	45.1	46.3	47.3	45.5	46.0	
	High	2.7	2.6	2.8	2.7	2.7	
Country of birth (%)	Sweden	79.9	79.3	79.9	79.8	79.6	
	Nordic country (excl. Sweden)	2.6	3.0	2.8	2.9	2.9	0.867^c^
	Europe (excl. Nordic countries)	7.0	7.8	7.3	7.4	7.6	
	Other	10.4	9.9	10.0	9.9	9.9	
	Missing	0.0	0.0	0.0	0.0	0.0	
Charlson Comorbidity Index (%)	0	85.1	83.9	83.5	84.4	84.2	0.450^c^
	1	10.5	11.7	11.9	11.3	11.4	
	>1	4.4	4.4	4.6	4.3	4.4	
TRD patients identified by other definitions (%)	0–Exclusively identified by this definition	-	0.0	0.5	34.1	16.0	<0.001^c^
	1 –By one other definition	-	1.8	0.1	1.4	2.4	
	2 –By more than one other definition	-	30.4	7.0	18.3	24.5	
	3 –By all definitions	-	67.7	92.4	46.2	57.2	
Days until TRD^b^	Mean (SD)	-	78.2 (122.8)	83.7 (123.5)	73.3 (132.3)	75.2 (121.3)	<0.001^d^
	Median (IQR)	-	69 (0–161)	75 (0–168)	60 (0–152)	64 (0–158)	

^a^ ‘Low’ income is defined as below the second decile (20) in the general population, ‘Average’ is between the second and the eight decile (20–80) of the general population and ‘High’ is above the eight percentile.

^b^ Days from index date to being defined as a TRD. The index date is the date the patients had had both first filling of the antidepressant and a depression diagnosis; this is the explanation why a small number of patients got 0 days before TRD and not 3 X required length of treatment in the model.

^c^ Chi-square test.

^d^ One-way ANOVA.

The models identified patients with similar mean age, ranging from 41.1 (MGH-s) to 42.4 (ESM). Annual income was also similar across the different models. The mean time from inclusion until being identified as having TRD ranged between 73.3 days for MGH-s to 83.7 days for ESM (p <0.001). Characteristics that did not differ between the groups were sex distribution (p = 0.995), country of birth (p = 0.867) and CCI (p = 0.450).

Table 2 shows a comparison of the four TRD models regarding the two clinical outcomes: proportion of patients experiencing psychiatric hospitalization within one year after being identified with TRD, and mortality from external cause of death. The rate of psychiatric hospitalization ranged between 16.6% in the MGH-s group to 21.6% in the ESM group. Compared with TRD patients identified by KIM, a lower risk for psychiatric hospitalization was found for patients in the MSM group (adjusted HR 0.95, 95% CI 0.94–0.97) and the MGH-s group (adjusted HR 0.92, 95% CI 0.90–0.94), whereas that of patients in the ESM group was marginally increased (adjusted HR 1.03, 95% CI 1.00–1.05). The mortality due to external causes ranged between 1.7% in the MGH-s group to 2.0% in the ESM group. The patients identified with TRD by the MGH-s had a reduced mortality by external causes compared with those identified by the KIM (adjusted HR 0.84, 95% CI 0.72–0.98), whereas no difference was found when comparing ESM and MSM with KIM. No substantial interaction effects could be identified between the four TRD models and the covariates (age, sex, country of birth, yearly income and CCI) on psychiatric hospitalization in a year after TRD and mortality from external cause of death (S1 and S2 Figs).

**Table 2:** Time to psychiatric hospitalization or death (external causes) comparing the Karolinska Institutet model (KIM) with the Massachusetts General Hospital Staging Method (MGH-s), the European Staging Method (ESM) and the Maudsley Staging Method (MSM).

				Unadjusted Cox regression		Adjusted Cox regression^a^	
TRD definition	Number	Events	Total person time, in years	HR	95% CI	HR	95% CI
**Psychiatric hospitalization in a year after TRD**^a^							
KIM	16,453	3,420 (20.8%)	9,885	1.00	Reference	1.00	Reference
ESM	12,059	2,608 (21.6%)	7,096	1.02	(0.98–1.04)	1.03	(1.00–1.05)
MGHs	24,112	3,995 (16.6%)	16,211	0.94	(0.92–0.96)	0.92	(0.90–0.94)
MSM	19,486	3,638 (18.7%)	12,033	0.96	(0.95–0.98)	0.95	(0.94–0.97)
**Mortality from external cause of death**							
KI-TRD	16,453	316 (1.9%)	60,711	1.00	Reference	1.00	Reference
ESM	12,059	243 (2.0%)	43,894	1.05	(0.88–1.24)	1.04	(0.88–1.23)
MGHs	24,112	412 (1.7%)	91,384	0.82	(0.71–0.96)	0.84	(0.72–0.98)
MSM	19,486	352 (1.8%)	72,877	0.93	(0.80–1.08)	0.95	(0.82–1.09)

^a^ The patient is categorized as a TRD patient from the date when all the criteria in each model is fulfilled *and* the patient has got a diagnosis of depression.

^b^ Adjusted by sex and age.

HR = Hazard Ratio;

TRD = treatment-resistant depression.

## 4. Discussion

In this study we found that the proportion of patients with depression who were identified with TRD differed when comparing various clinical models adapted for clinical use with the KIM, ranging between 9.5% to 19.0%. We also found that the model identifying the highest proportion of patients with TRD, the MGH-s, identified a TRD group with somewhat lower risk for psychiatric hospitalization and risk for mortality from accidents and suicide than the other models, implying that a wider selection includes patients with lower disease burden.

### 4.1. Comparison between models

Although differences were statistically significant, proportions of TRD identified across the models were comparable, and the sociodemographic characteristics of the patients similar. This is also in line with the finding that no major interaction effects could be identified between the models and the covariates on the two study outcomes. As the comparisons are made in a large dataset, even small differences can reach statistical significance. The difference in the proportions of TRD observed may be due to the differing clinical criteria in the models, with the highest impact most likely coming from the parameters of treatment time required, as well as dosage increase and add-on medication. The most conservative model, the ESM, requires 12–16 weeks of continuous treatment for an adequate treatment trial, and subsequently included about half as many TRD patients as the MGH-s, which only requires six weeks as continuous treatment while also adding points towards TRD for every dose increase and add-on medication tried.

As register data seldom contain information on clinical variables, the KIM is based on prescription fill data and recorded diagnoses alone. Another main feature of the KIM is that 28 days is used as the lower limit required for an adequate treatment trial. This much shorter duration is most likely the reason for yielding higher proportions than the ESM (12–16 weeks). Regarding the parameter of increasing the dosage above the recommended starting dose, this was not included in the KIM as it is in general does not increase likelihood of treatment response [28].

### 4.2. Comparison with previous research

The comparison between the proportions of TRD identified in this study with those in previous register studies is made difficult by varying study populations and selection methods; e.g. the present study being the only one requiring patients to have been diagnosed with MDD in specialized healthcare. However, results are similar to proportions found in similar register data in Taiwan and in US claims data (16% and 12% respectively) [7, 12]. Other register based studied have taken into account the number of dose titrations [7] and number of prescription fills [8] as measures of treatment optimization. Treatment length in this study was estimated counting tablets rather than number of prescription fills, which may have improved the accuracy.

Proportions of TRD are consistently lower in register studies than in clinical studies, where the proportion of patients not achieving remission or response has been reported to be as high as 50% [1, 2]. Differences are likely due to the impact of the close clinical monitoring that participation in clinical studies entails with fewer patients lost to follow-up.

### 4.3. Strengths and limitations

The strengths of this study include the use of register data with nationwide coverage and high completeness, yielding a large study population. The prescription fill information in the PDR combined with manual extraction of text information granted high granularity in constructing treatment episodes.

There are also weaknesses, among which the lack of clinical data, e.g. from patients’ records, may be the most important. Attempts to estimate TRD from patients’ prescription fills of antidepressants without insight into the prescribing doctor’s clinical intention will always be uncertain. Some patients may have no changes in the treatment regime despite lack of response, while others will have changes for other reasons such as side effects or administrative reasons, e.g. medication costs. Additionally, antidepressant drugs are also used for other indications than major depression, such as anxiety disorders or premenstrual syndrome. Because the NPR does not include diagnostic data from primary care, the MDD cohort was restricted to patients with a diagnosis of depression from the specialized psychiatric care. This excluded patients who underwent all treatment trials in primary care but may have increased the specificity of the diagnosis.

Also, register adaptations of clinical models are bound to have limitations, and several parameters in the clinical models could not be readily transferred in the data available. The MSM includes a dimension of duration of the depressive episode that could not be included in our adaptation. In the adaptation of the ESM we failed to find a way to detect dose and duration optimization with high accuracy from prescription fill data. Neither the MGH-s model nor the ESM model were originally intended to be used as threshold measurements as we do here, although they have been used for this purpose previously [29].

Finally, the patients’ overlap between the KIM and the other TRD definitions may violate the assumption of independence in the Cox regressions. However, the results from the Cox regressions appear to be valid, since the results reflect the differing patient characteristics identified by the definitions (e.g. the higher number of patients identified by the MGH-s in turn yielding lower HRs for the clinical outcomes).

### 4.4. Clinical significance

Models for identifying TRD in registers—including the KIM—may have several applications in register-based research provided that the clinical validity is acceptable. Although TRD is a common condition with potentially detrimental consequences, there is a relative lack of knowledge regarding both risk factors for developing TRD and on longitudinal outcomes among patients with TRD [30–32]. Proportions of TRD in register data differ depending on which definition is used, most likely because of the varying clinical parameters included. A register-based approach to identify TRD patients, such as the KIM, manages to identify patients with clinical characteristics like hospitalization typical of TRD, but does not suffer from the limitations of the other models which could include overlong treatment duration not consistent with clinical practice. Against the background knowledge regarding the clinical characteristics of the patients identified by various models, the present study suggests that the KIM may be a useful tool for register-based research of TRD.

## 5. Conclusion

The differing clinical parameters included in clinical models of TRD may lead to varying proportions of TRD patients identified when adapted for register research. The KIM model of defining TRD tailored for register-based research captures patients with characteristics comparable to those which are captured by adaptations of the commonly used clinic-based definitions. Thus, models for identifying TRD specifically designed for register use, not depending on clinical data, may identify similar patients, and could be applied in future register-based research furthering the understanding of this group of patients.

## Supporting information

S1 Fig Interaction effects between the four TRD models and the covariates (sex, age, CCI, country of birth and yearly income) on psychiatric hospitalization in a year after TRD. (TIFF)

S2 Fig Interaction effects between the four TRD models and the covariates (sex, age, CCI, country of birth and yearly income) on mortality from external cause of death estimated by Cox regression. (TIFF)

S1 Table Definition of adequate dose for the respective antidepressant drugs. (PDF)

## Abbreviations

AD: Antidepressant
aHR: Adjusted hazard ratio
CCI: Charlson comorbidity index
CDR: The Swedish Cause of Death Register
CI: Confidence intervals
CRD: Chronic resistant depression
ECT: Electroconvulsive treatment
ESM: European Staging Model
HR: Hazard ratio
ICD: International Statistical Classification of Diseases and Related Health Problems
KIM: Karolinska Institutet Model
LISA: The Longitudinal Integration Database for Health Insurance and Labor Market Studies
MDD: Major depressive disorder
MGH-s: Massachusetts General Hospital Staging Method
MSM: Maudsley Staging Model
NPR: The National Patient Register
PDR: The Prescribed Drug Register
rTMS: Repetitive transcranial magnetic stimulation
TRD: Treatment-Resistant Depression
