# Supplementary figures and images for: A register-based approach to identifying treatment-resistant depression—Comparison with clinical definitions
Source: PLoS One. 2020 Jul 30;15(7):e0236434. doi: 10.1371/journal.pone.0236434 (PMC7392234; doi:10.1371/journal.pone.0236434)

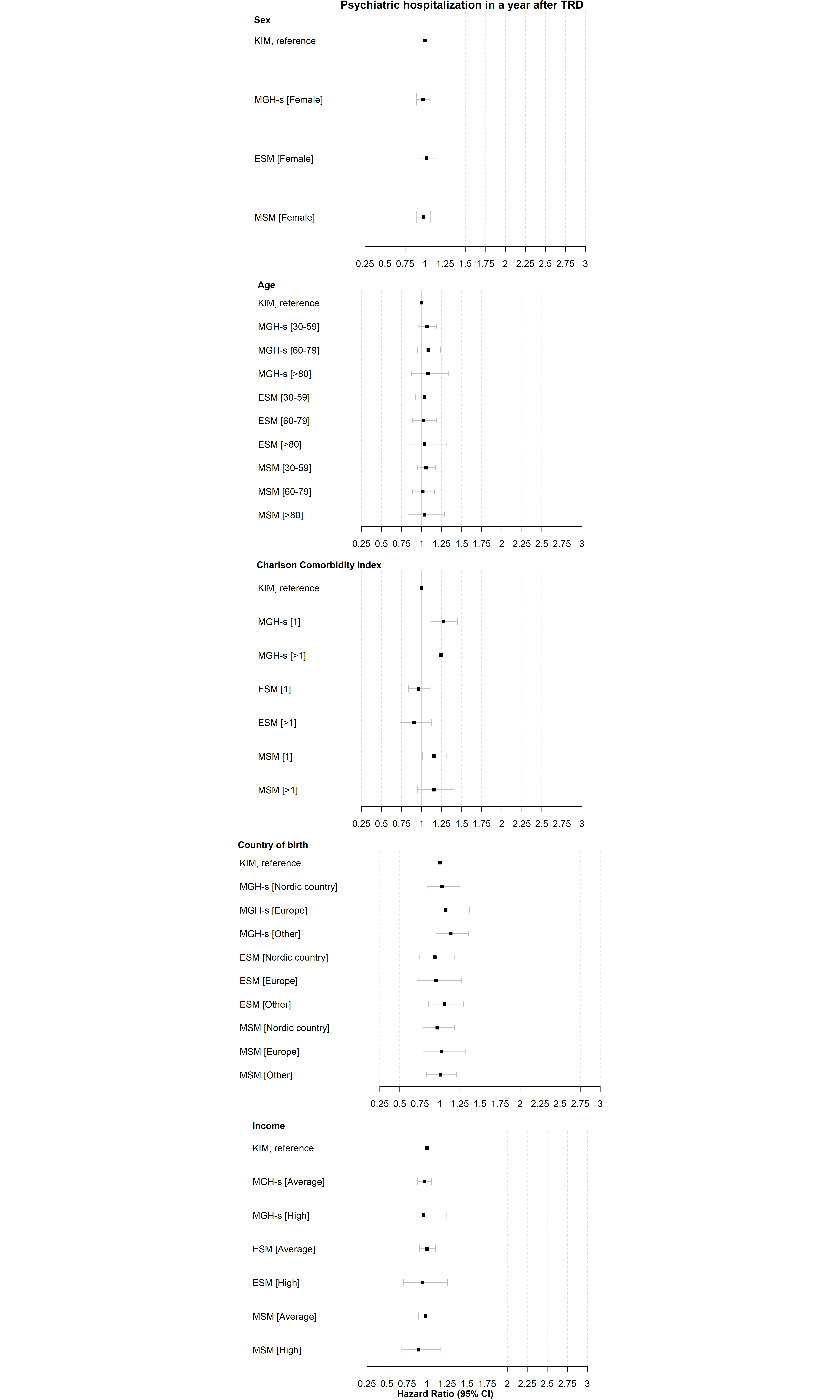

Supplement: S1 Fig — (TIFF) [file pone.0236434.s001.tiff]

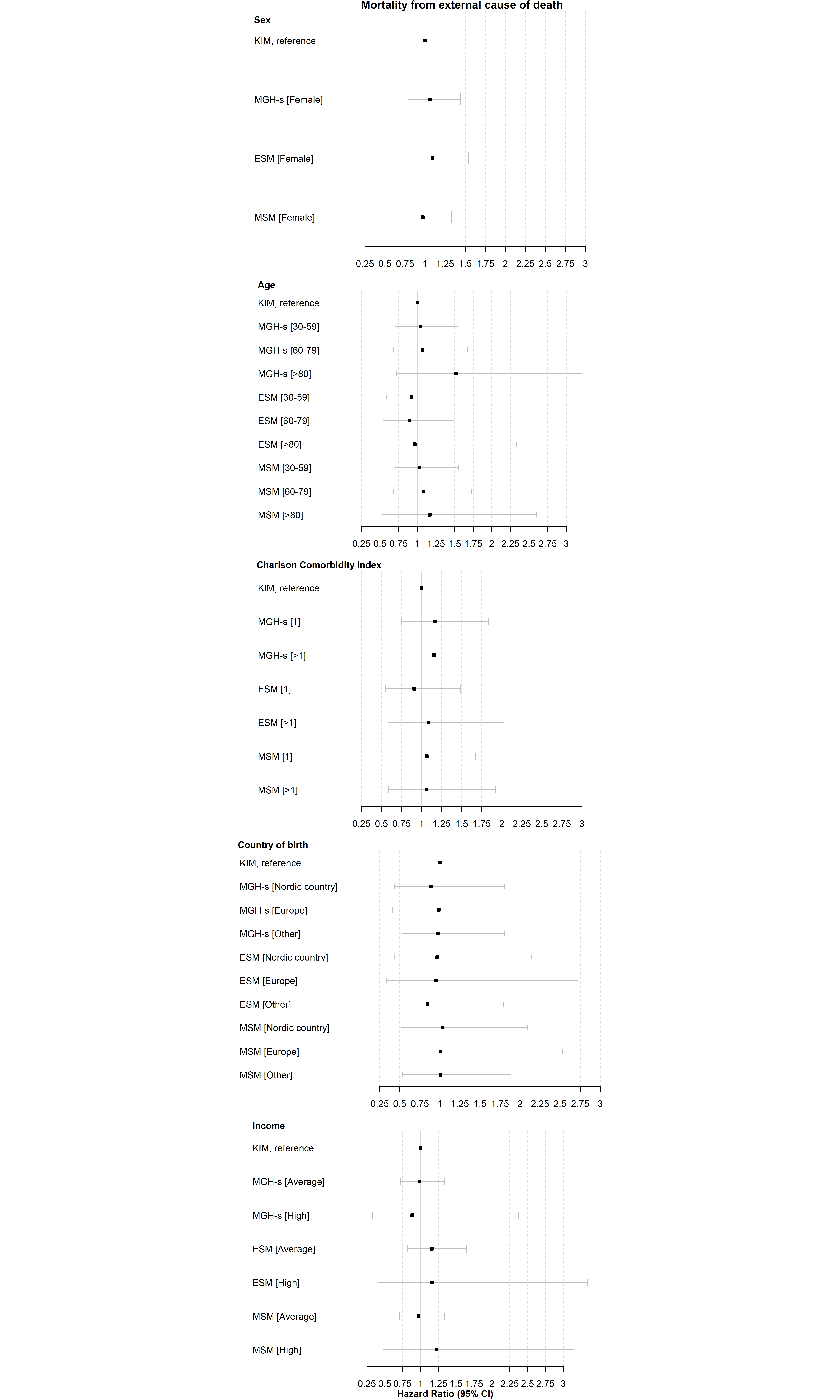

Supplement: S2 Fig — (TIFF) [file pone.0236434.s002.tiff]
