# Supplementary material for: A register-based approach to identifying treatment-resistant depression—Comparison with clinical definitions
Source: PLoS One. 2020 Jul 30;15(7):e0236434. doi: 10.1371/journal.pone.0236434 (PMC7392234; doi:10.1371/journal.pone.0236434)
Supplement: S1 Table — (PDF) [file pone.0236434.s003.pdf]

ATC	Name	DDD (mg)	Lowest Dose (mg)	Adequate Dose (mg)
N06AA02	imipramine	100	50	100
N06AA04	clomipramine	100	50	75
N06AA06	trimipramine	150	50	75
N06AA09	amitriptyline	75	75	75
N06AA10	nortriptyline	75	75	75
N06AA21	maprotiline	100	75	75
N06AB03	fluoxetine	20	20	20
N06AB04	citalopram	20	20	20
N06AB05	paroxetine	20	20	20
N06AB06	sertraline	50	50	50
N06AB08	fluvoxamine	100	100	100
N06AB10	escitalopram	10	10	10
N06AF03	phenelzine	60	60	90
N06AF04	tranylcypromine	10	30	30
N06AG02	moclobemide	300	300	300
N06AX03	mianserin	60	30	30
N06AX05	trazodone	300	150	150
N06AX06	nefazodone	400	300	400
N06AX11	mirtazapine	30	30	30
N06AX12	bupropion	300	150	150
N06AX14	tianeptine	37.5	25	25
N06AX16	venlafaxine	100	75	75
N06AX17	milnacipran	100	100	100
N06AX18	reboxetine	8	8	8
N06AX21	duloxetine	60	60	60
N06AX22	agomelatine	25	25	25
N06AX26	vortioxetine	10	10	10
